# Functional Characterization of a Trehalose-6-Phosphate Synthase in *Diaphorina citri* Revealed by RNA Interference and Transcriptome Sequencing

**DOI:** 10.3390/insects12121074

**Published:** 2021-11-30

**Authors:** Jian-Chun Song, Zhan-Jun Lu, Long Yi, Hai-Zhong Yu

**Affiliations:** 1 College of Life Science, Gannan Normal University, Ganzhou 341000, China; jianchunsong951@163.com (J.-C.S.); luzhanjun7@139.com (Z.-J.L.); 2 National Navel Orange Engineering Research Center, Ganzhou 341000, China

**Keywords:** *Diaphorina citri*, trehalose-6-phosphate synthase, RNA interference, transcriptome sequencing

## Abstract

**Simple Summary:**

Trehalose-6-phosphate synthase (TPS) is a key enzyme in regulating trehalose content in the insect hemolymph. The loss or dysfunction significantly affects the growth and development of insects. *Diaphorina citri* is a notorious phloem sap-sucking pest that can spread huanglongbing between the diseased tree and the healthy tree. The control of huanglongbing mainly depends the management of *D. citri*. So far, the management of *D. citri* populations has depended on using chemical pesticides, though pesticide abuse has caused serious problems. Therefore, it necessary to find new targets for *D. citri* control. In this paper, we identified a *TPS* gene from *Diaphorina citri*, and named it *DcTPS1*. Silencing of *DcTPS1* induced an abnormal phenotype, and inhibited chitin metabolism and fatty acid metabolism. Moreover, the mortality and malformation rate significantly increased, and the molting rate decreased after inhibition of *DcTPS1*. KEGG analysis revealed that upregulated DEGs were mainly responsible for oxidative phosphorylation, whereas downregulated DEGs were mainly related to lysosome and ribosome. Overall, our data suggested that *DcTPS1* might play a crucial role for the growth and development of *D. citri*.

**Abstract:**

Trehalose-6-phosphate synthase (TPS) plays an important role in the synthesis of trehalose. In the current study, a *TPS* gene was obtained from *Diaphorina citri*, and named as *DcTPS1* which encoded a protein of 833 amino acid residues. Real-time quantitative PCR (qPCR) analysis revealed that *DcTPS1* had the highest expression level in the midgut and fifth-instar nymph stage. Knockdown of *DcTPS1* by RNA interference (RNAi) induced an abnormal phenotype and increased mortality and malformation rate with a decreased molting rate. In addition, silencing of *DcTPS1* significantly inhibited *D. citri* chitin metabolism and fatty acid metabolism, while the expression levels of fatty acid decomposition-related genes were downregulated. Furthermore, comparative transcriptomics analysis revealed that 791 differentially expressed genes (DEGs) were upregulated and 678 DEGs were downregulated when comparing ds*DcTPS1* groups with ds*GFP* groups. Bioinformatics analysis showed that upregulated DEGs were mainly involved in oxidative phosphorylation, whereas downregulated DEGs were mainly attributed to the lysosome and ribosome. These results indicated that *DcTPS1* played an important role in the growth and development of *D. citri*.

## 1. Introduction

Trehalose is a nonreducing disaccharide which is widely distributed in various organisms, including bacteria, fungi, insects, invertebrates and plants [1]. In insects, trehalose exists mainly in the hemolymph, and plays an important role as an instant energy source, facilitating carbohydrate absorption, and as a starting substrate for chitin biosynthesis [2,3,4]. In insects, biosynthesis of trehalose is catalyzed by trehalose-6-phosphate synthase (TPS) and trehalose-6-phosphate phosphatase (TPP), and then is transported across the cellular membrane into the hemolymph through trehalose transporter [5,6]. To date, *TPS* genes have been identified from different insect species, and play crucial functions in regulating insect sugar metabolism, chitin metabolism, and stress reactions [7,8,9,10,11]. However, the *TPP* genes have not been found in many insect species, but *TPS* genes that encode proteins with both TPS and TPP domains were identified in the same species [12]. In *Drosophila*, a mutation of the *TPS1* gene failed to produce trehalose and exhibited severe growth defects on a low-protein diet [13]. Shi et al. revealed that knockdown of *Leptinotarsa decemlineata TPS* (*LdTPS*) gene decreased trehalose and chitin content [10]. In *Dendroctonus ponderosae*, *TPS* expression levels are high in the autumn, while they are significantly lower in the spring. The results indicated that TPS is mainly involved in the synthesis of trehalose for *D. ponderosae* survival during cold periods [14]. In previous research, Liu et al. characterized a *TPS* gene from *Diaphorina citri*, and silencing of *DcTPS* by RNAi significantly reduced trehalose content [15]. However, the specific biological functions of *TPS* gene in *D. citri* are still unclear.

*Diaphorina citri* is a phloem sap-sucking insect which feeds on citrus and is distributed worldwide [16]. *D. citri* nymphs and adults feed on phloem sap of rutaceous plants, consequently cause leaf distortion, curling and yellowing [17]. Furthermore, because the phloem sap is rich in various sugars, *D. citri* excretes large amounts of honeydew causing citrus fuliginous disease [18]. Additionally, *D. citri* transmits the bacteria *Candaidatus* Liberbacter asiaticus, causing huanglongbing (HLB). HLB is a plant disease, which causes serious losses for the citrus industry every year [19]. Nowadays, chemical pesticides play a dominant role in the prevention and control of *D. citri*, including pyrethroid, organophosphate, and neonicotinoid classes [20]. Aggressive application of insecticides has caused many problems, such as environment pollution, human poisoning and insecticide resistance [21,22]. Therefore, there is a need for treatments with new modes of action for the control of *D. citri*, such as RNAi.

RNAi has been developed as an useful tool for functional gene research, which was triggered by double-stranded RNA (dsRNA) [23,24]. Therefore, RNAi has shown great potential for pest control [25]. The RNAi mechanism was described for the first time in the nematode, following which it was discovered to be a common phenomenon in eukaryotic organisms, including protozoans, invertebrates, vertebrates, fungi, algae and plants [26]. However, many factors limiting RNAi efficiency has been reported, such as incomplete dsRNA internalization, instability of dsRNA, impaired systemic spreading of the RNAi signal, and refractory target genes [27]. Therefore, efficient dsRNA delivery method and selection of appropriate target genes are essential prerequisites for pest management. Additionally, concentration and length of dsRNA are further factors contributing to a change in RNAi penetrance [28]. RNAi has been successfully applied to research the gene functions in various insects, including lepidopteran, hemipteran, coleoptera and dipteran [29,30,31]. In *D. citri*, for different target genes, the dsRNA delivery methods need to be changed accordingly. Yuan et al. revealed that silenced *D. citri NADPH-cytochrome P450 reductase* (*DcCPR*) increased the sensibility of *D. citri* to imidacloprid and thiamethoxam by RNAi using the parafilm feeding method [32]. According to topical feeding with dsRNA-*AChE*, the sensitivity of *D. citri* nymphs to chlorpyrifos and carbaryl was significantly increased [33]. Using an artificial diet mixed with dsRNA, knockdown of *DcGSTd1* and *DcGSTe2* significantly increased the mortalities of thiamethoxam-treated psyllid [34]. Using to the parafilm feeding method, silencing of *DcCHS* significantly reduced the expression levels of chitin metabolism-related genes, which were significantly downregulated [5].

In the present research, we identified a *TPS* gene from *D. citri* genome database. qPCR was performed to analyze the expression profiles of *DcTPS1* in different tissues and at different developmental stages. In addition, the functions of *DcTPS1* were described by RNAi and transcriptome sequencing. These results will provide a new target for further control of *D. citri*.

## 2. Materials and Methods

### 2.1. Insect Rearing and Sample Prepartion

The *D. citri* were obtained from a citrus germplasm resource nursery located in Gannan Normal University, Ganzhou, China. The *D. citri* were continuously reared using *Murraya exotica*. The rearing conditions were 26 ± 1 °C, 60 ± 5% relative humidity under a 14:10 dark light cycle. To keep the consistency of *D. citri* growth, the mated *D. citri* females were released into the flourishing *Murraya exotica* with many bud breaks in an insect rearing cage. After 48 h, all *D. citri* adults were removed using a portable aspirator. According to morphological features (the length of cohort size from egg to fifth-instar nymph stage are 0.253, 0.304, 0.46, 0.675, 1.038 and 1.563, respectively), *D. citri* at seven different stages were collected under a stereomicroscope with a camel hairbrush. The psyllid eggs were harvested using a sterilized blade, and then pooled together. All collected samples were kept at −80 °C. Each group of samples contained three biological replicates.

### 2.2. Cloning of DcTPS1 and Bioinformatic Analysis

The sequence of *TPS* genes from *Nilaparvata lugens*, *Acyrthosiphon pisum* and *Drosophila melanogaster* were downloaded and used for blasting against the *D. citri* genome database. After sequence assembly, splicing and sequencing, *DcTPS1* with a complete open reading frame was identified and amplified according to PCR. The purified product was linked to pMD19-T and sequenced by a biotechnology company (Sangon Biotech, Shanghai, China).

The amino acid sequence of DcTPS1 was analyzed by using DNASTAR software. The molecular weight (MW) and isogenic point (pI) were predicted by ExPASy (http://web.expasy.org/compute_pi (accessed on 1 May 2021)). The structural domain was identified by using SMART online software (http://smart.embl-heidelberg.de/ (accessed on 20 May 2021)). The phylogenetic tree was constructed using MEGA 7.0 with the neighbor-joining method with 1000 replicates. In addition, the glycosylation sites were predicted according to NetNGlyc 1.0 Server (https://www.cbs.dtu.dk/services/NetNGlyc/ (accessed on 1 May 2021)).

### 2.3. dsRNA Synthesis and DcTPS1 RNA Interference Analysis

For RNA interference, specific primers of *DcTPS1* (accession No: MZ888936) and *GFP* (accession No: X83959) with T7 promoters were designed and are presented in Table S1. The ds*DcTPS1* and ds*GFP* were synthesized using the T7 RioMAX^TM^ Express RNAi System (Promega, CarIsbad, CA, USA), and the delivery of dsRNA was performed based on a previous protocol [5]. In brief, the length of the amplified sequence for ds*DcTPS1* synthesis is 462 bp (ds*DcTPS1* fragment from 1419 bp to 1880 bp), and the purified products were linked to pMD19-T for obtaining the recombinant plasmid. The target sequence was further amplified using primers with T7 promoters, and then added to RiboMAXTM Express T7 2× Buffer, nuclease-free water and Enzyme mix T7 express. The mixture was incubated for 4 h at 37 °C and 10 min at 70 °C. The RNase solution and RQ RNase-free DNase were added and incubated for 30 min at 37 °C. Finally, the synthetic ds*DcTPS1* was diluted to 500 ng/µL using RNase-free water containing 15% sucrose and 0.1% red food dye and added between two layers of stretched parafilm which was fixed on a glass double pipe. In total, 180 fifth-instar *D. citri* nymphs were divided into three groups and placed to the glass double pipe for 24 h, and then transferred onto the fresh *M. exotica* seedlings. All experiments contained three biological replicates. All the living *D. citri* were collected at 24 h and 48 h after dsRNA treatment. The effects of ds*DcTPS1* on gene expression were analyzed using qPCR.

### 2.4. qPCR Analysis of DcTPS1

The total RNA was extracted from *D. citri* at different tissues (head, leg, midgut, fat body and wing) and nymph at different instars (egg, first-, second-, third-, fourth-, fifth-instar nymphs) using TRIzol reagent (Invitrogen). All samples consisted of three biological replicates. The purity and concentration of RNA were measured by a spectrophotometer (NanoDrop 2000, Thermo Fisher Scientific, New York, NY, USA). The cDNA was synthesized using the PrimeScript^TM^ RT Reagent Kit with gDNA Eraser (TaKaRa, Dalian, China) based on a previous protocol.

qPCR was conducted to analyze the expression levels of DcTPS1 in different tissues and developmental stages. The primers are presented in Table S1. The reaction procedures were set as follows: 40 cycles at 95 °C for 10 s and 60 °C for 10 s. The reactions were performed with a LightCycler^®^ 96 PCR detection system (Roche, Basel, Switzerland). The relative expression levels were calculated using 2^−ΔΔCt^ method. The reference gene was *glyceraldehyde-3-phosphate dehydrogenase* (*GAPDH*). All experiments contained three biological replicates.

### 2.5. cDNA Libaray Preparation and Illumina Sequencing

The cDNA library preparation and Illumina sequencing were performed at Novogene Biological Information Technology Co., Ltd. (Tianjin, China). Approximately 50 *D. citri* were collected from each treatment group (treated with ds*DcTPS1*) and control groups (treated with ds*GFP*) at 48 h after ingestion of dsRNA. All experiments contained three biological replicates. RNA concentration and purity were measured according to a Qubit RNA Assay Kit in a Qubit^®^2.0 Fluorometer (Life Technologies, CA, USA). In total, 1 µg RNA was used to construct cDNA library by TruSeq RNA Sample Preparation Kit v2 (Illumina, San Diego, CA, USA) according to the manufacturer’s instructions.

The prepared cDNA library was sequenced by the Illumina HiSeq platform, generating 150 bp paired-end reads. The clean reads were obtained by removing reads containing the adapter from raw data. Additionally, the Q20, the Q30 and the GC-content of the clean data were calculated.

### 2.6. Transcriptome Analsysis after Silencing of DcTPS1

The transcriptome data were mapped to *D. citri* reference genome (ftp://ftp.citrusgreening.org/annotation/OSGv2.0 (accessed on 1 January 2021)) using Hisat2 (version 2.0.5; https://anaconda.org/biobuilds/hisat2 (accessed on 1 January 2021)) aligner. This generated a database of splice junctions based on the gene model annotation file. The expression levels of these genes were calculated using reads per kilobase of exon per million reads mapped. Differential expression analyses of genes between dsGFP and dsDcTPS1 groups were performed using the DESeq2 R package. *p*-values were adjusted using the Benhamini-Hochberg method to control for the false-discovery rate. A corrected *p*-value of 0.05 and an absolute |log2 (fold change)| (Fold change > 1) of 0 were set as the thresholds for significantly different gene expression. The hierarchical cluster analysis of DEGs was conducted using Genesis software (http://genome.tugraz.at/genesisclient_download.shtml (accessed on 1 January 2021)).

Gene ontology (GO) is a tool used for gene annotation by collecting a defined, structured and controlled vocabulary. The topGO R package, which implements the GO terms, was used for the enrichment analysis of length-corrected DEGs. Kyoto Encyclopedia of Genes and Genomes (KEGG) is a database that can be used to understand the high-level functions and utilities of biological systems, such as cells, organisms and ecosystems from molecular level. A KEGG pathway enrichment analysis for DEGs was performed using KOBAS. A *p*-value of <0.01 was set as the threshold.

## 3. Results

### 3.1. Analysis of the cDNA and Protein Sequence of DcTPS1

**Figure 1:**
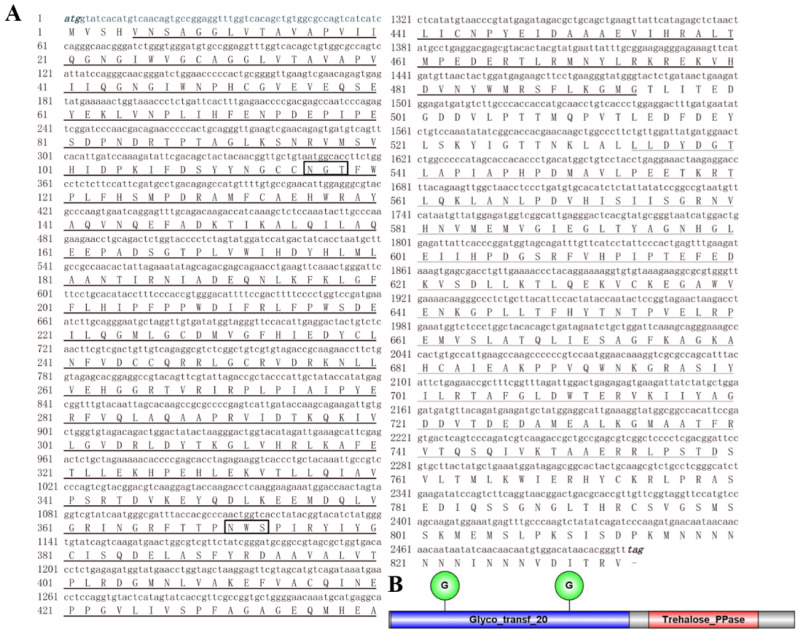
Sequence analysis of *DcTPS1* cDNA sequence. (**A**) Nucleotide and amino acid sequence analysis of the *DcTPS1* cDNA sequence. Numbers on the left side indicate the position of nucleotide and amino acid. The initiation codon (ATG) and termination codon (TAG) are showed in bold italics. The black solid line indicates the Glyco_transf_20 domain. The black break line indicates the Trehalose_PPase domain. The black box represents two glycosylation sites. (**B**) Structural domain analysis of DcTPS1 by SMART software. The blue box represents Glyco_transf_20 domain. The red box represents Trehalose_PPase domain. Two green circles indicate the glycosylation sites.

**Figure 2:**
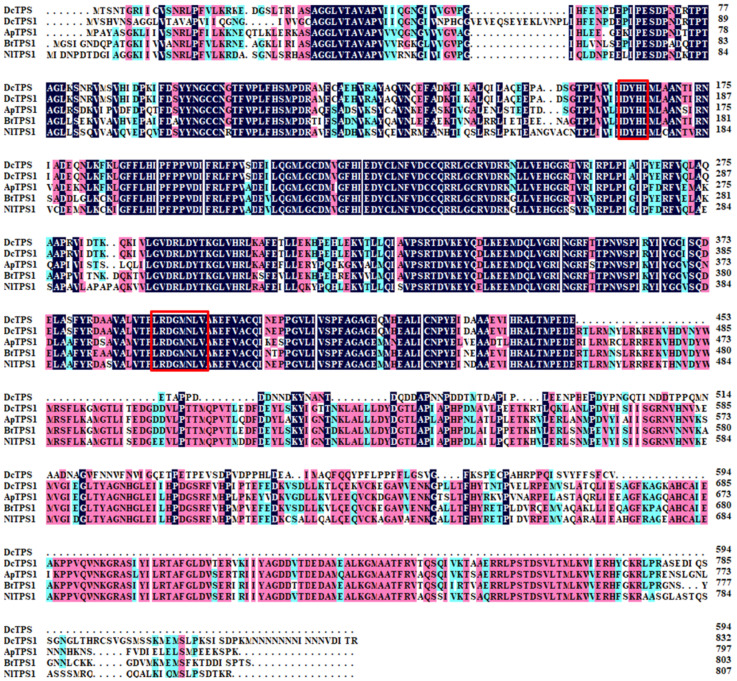
Multiple sequence alignment of the conserved domain of the TPS1 from four insect species, including *Diaphrina citri* TPS1 (MZ888936), *Diaphorina citri* TPS (QOU11567), *Acyrthosiphon pisum* TPS1 (XP_001945523), *Bemisia tabaci* TPS1 (XP_018916964) and *Nilaparvata lugens* TPS1 (ACV20871). Signature motifs (HDYHL and DGMNLV) unique to TPS was presented with red box. The conserved amino acid residues are highlighted in black, and similar amino acid residues are labelled in pink and blue.

A TPS gene was identified and named *DcTPS1* (GenBank accession:MZ888936). Bioinformatic analysis revealed that the ORF of *DcTPS1* is 2502 bp, encoding a protein with 833 amino acids. The predicted MW is 93.60 kDa and pI is 5.73 (Figure 1A). SMART software analysis suggested that DcTPS1 contained one Glyco_transf_20 domain (5–494) and one Trehalose_PPase domain (534–759) (Figure 1B). The multiple sequence alignment of TPSs form different insects revealed that DcTPS1 protein sequence had 74.38%, 71.60% and 70.84% identities with those of *B. tabaci*, *A. pisum* and *N. lugens*, respectively, and two signature motifs of HDYHL (174–178) and DGMNLV (404–409) were also found (Figure 2). In addition, multiple sequence alignment analysis suggested that DcTPS1 protein sequence had 53.83% identifies with DcTPS protein and exhibited a significant difference at the C-terminal. The *DcTPS1* gene sequence had 58.77% similarities to the *DcTPS* gene sequence (Figure 2). In addition, there were two glycosylation sites (NGT and NWS) found in DcTPS1 (Figure 1A). The phylogenetic analysis showed that TPS can be divided into two categories, including TPS1 and TPS2. DcTPS1 had a close relationship with the TPS1 of sap-sucking hemipteran, *B. tabaci*, *A. pisum* and *N. lugens*, but it kept a distant relationship with the TPS2 (Figure S1).

### 3.2. Tissue Distribution and Developmental Stages Expression Patterns of DcTPS1

**Figure 3:**
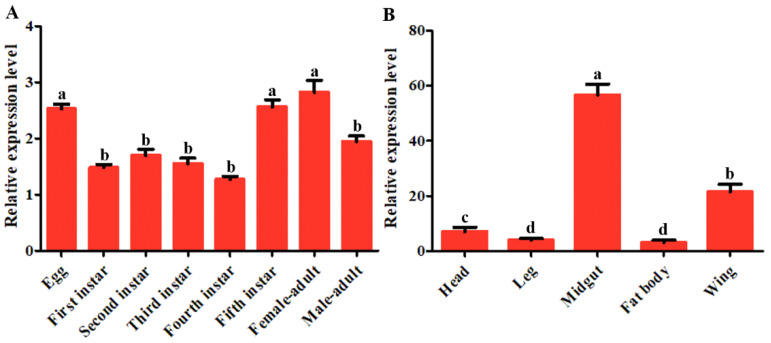
The spatiotemporal expression patterns of *DcTPS1* in different developmental stages (**A**) and different tissues (**B**) of *Diaphorina citri*. Relative expression levels of *DcTPS1* were analyzed using qPCR. Data were normalized using *glyceraldehyde-3-phosphate dehydrogenase* (*GAPDH*) and are represented as the means ± standard errors of the means from three independent experiments. The 2^−∆∆Ct^ method was used to calculate the relative expression level. Statistical analysis was conducted using SPSS software. Different letters indicate significant differences, for example, a, b, c and d (*p* < 0.05).

The expression patterns of *DcTPS1* in different tissues and at different developmental stages were analyzed by qPCR. The results showed that the *DcTPS1* expression could be detected in all tissues, including head, leg, midgut, fat body and wing (Figure 3). Higher expression of *DcTPS1* was found in the midgut and wing tissues, whereas it had low expression levels in the leg and fat body (Figure 3). The expression level of *DcTPS1* in the midgut was 18.3 times of that in the fat body. Additionally, the expression level of *DcTPS1* was significantly downregulated from egg to nymph stages. However, the expression level of *DcTPS1* was constantly observed without significant differences from first-instar nymph to fourth-instar nymph (Figure 3). *DcTPS1* expression level was upregulated from the fourth-instar nymph to fifth-instar nymph stages. Interestingly, the expression levels of *DcTPS1* in the female adults were higher than in the male adults (Figure 3).

### 3.3. Analysis of Mortality, Molting and Malformation Rate after Inhibition of DcTPS1

**Figure 4:**
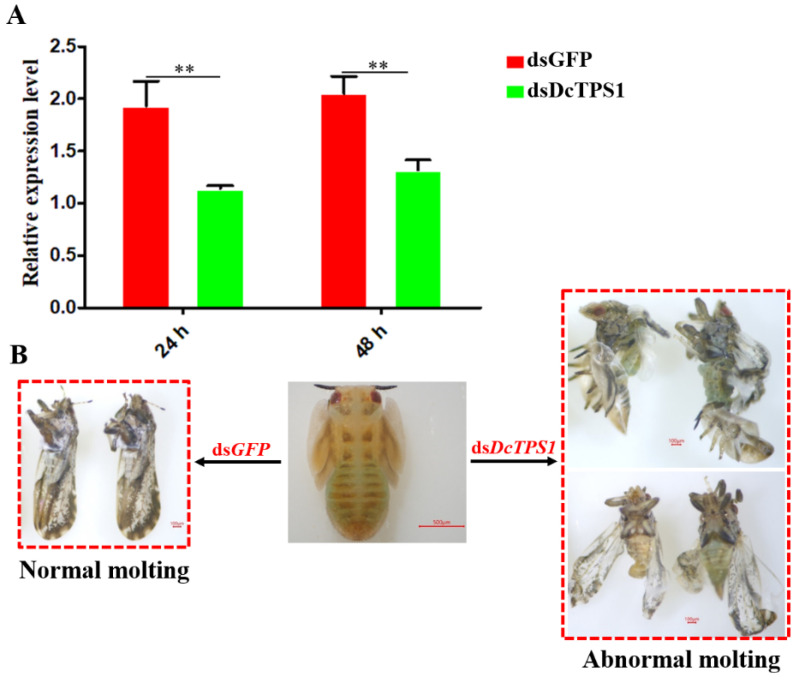
Detection of *DcTPS1* expression levels and phenotypic observation after treatment with ds*DcTPS1* and ds*GFP*. (**A**) Analysis of *DcTPS1* expression levels after treatment with ds*DcTPS1* and ds*GFP*. The 2^−∆∆Ct^ method was adopted to calculate the relative expression level. The SPSS software was used to conduct statistical analysis. The asterisks indicate the significance differences by ** *p* < 0.01. (**B**) Phenotypic observation of *D. citri* adult at 48 h.

RNAi was performed to determine the biological functions of *DcTPS1* in the development of *D. citri*. The results showed that *DcTPS1* expression level was significantly downregulated at 24 h and 48 h after ingestion of dsRNA (Figure 4A). However, the differences in expression levels between ds*DcTPS1* group and ds*GFP* group had no significant change at 24 h and 48 h. Regarding phenotype, the transition from fifth-instar nymph to adult was disrupted in the ds*DcTPS1* treatment group, and the emerged *D. citri* adult exhibited two abnormal phenotypes. Phenotypic observation showed that the treated nymphs molted into adults with abnormal dorsal tergites or malformed wings. Wings were irregular and curled at the distal end or smaller in size. Legs were curled and could not be stretched. Some nymphs failed to completely molt. However, in the ds*GFP* control group, the nymphs could molt normally (Figure 4B).

**Figure 5:**
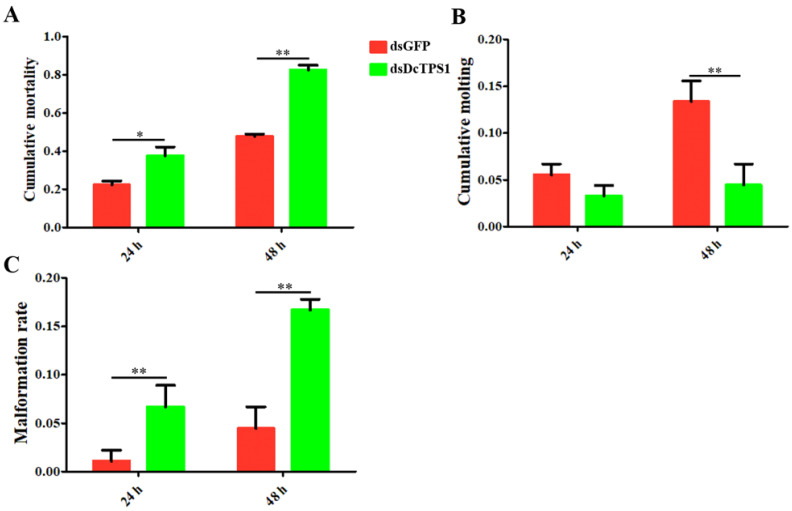
Statistical analysis of *D. citri* mortality, malformation and molting after inhibition of *DcTPS1*. (**A**) Determination of *D. citri* mortality after inhibition of *DcTPS1*. The ds*GFP* treatment group was used as a control; (**B**) Detection of malformation rate of *D. citri* at 24 h and 48 h after RNAi of *DcTPS1*; (**C**) Detection of cumulative molting of *D. citri* at 24 h and 48 h after inhibition of *DcTPS1*. The asterisks indicate the significance differences by * *p* < 0.05 and ** *p* < 0.01. The SPSS software was used to conduct statistical analysis.

The malformation rates and cumulative mortality significantly increased after silencing of *DcTPS1* at 24 h and 48 h. The cumulative mortality in the treatment group (ds*DcTPS1*) was 37.6%, while it was 22.2% in the control group (ds*GFP*) at 24 h, and the mortality reached 82.5% at 48 h (Figure 5A). The malformation rate in the ds*GFP* control group was 1.1%, and had no significant change from 24 hpt to 48 hpt, while the malformation rate in the ds*DcTPS1* treatment group increased from 6.7% to 16.7% during this period (Figure 5B). In contrast, for the rate of the cumulative molting between 24 hpt and 48 hpt, no significant difference was observed in the ds*DcTPS1*-treated *D. citri*, while it increased from 5.6% to 13.4% in the control group (Figure 5C). These results indicated that silencing of *DcTPS1* impaired the molting process of fifth-instar *D. citri* nymphs.

### 3.4. Analysis of the Effect on Chitin Metabolism after Silencing DcTPS1

**Figure 6:**
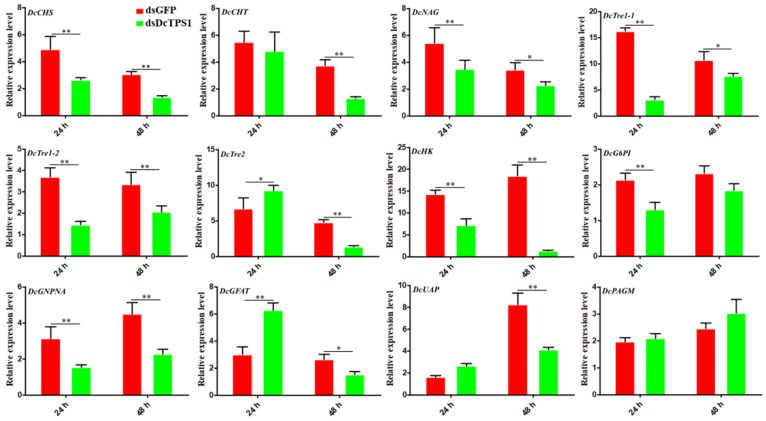
Analysis of expression levels of chitin metabolism-related genes after inhibition of *DcTPS1*. The ds*GFP* treatment group was used as a control. The mean expression level represents three biological replicates. The asterisks indicate significant differences by * *p* < 0.05 and ** *p* < 0.01. The SPSS software was used to conduct statistical analysis.

The results showed that the relative expression levels of *D. citri* chitin synthase (DcCHS), *D. citri* beta-*N*-acetylglucosaminidase (DcNAG), *D. citri* trehalose 1-1 (DcTre1-1), *D. citri* trehalose 1-2 (DcTre1-2), *D. citri* hexokinase (DcHK) and *D. citri* glucosamine-phosphate *N*-acetyltransferase (DcGNPNA) were downregulated at 24 h and 48 h after silencing of *DcTPS1* (Figure 6). The relative expression levels of *D. citri* chitinase (DcCHT) and *D. citri* UDP-*N*-acetylglucosamine pyrophosphorylase (DcUAP) were downregulated at 48 h after silencing of *DcTPS1*, but it had no obvious change at 24 h (Figure 6). Additionally, two genes (DcTre2 and *D. citri* fructose-6-phosphate transaminase (DcGFAT)) showed upregulation at 24 h after knockdown of *DcTPS1*, whereas their expression levels were downregulated at 48 h (Figure 6).

### 3.5. Analysis of the Effect on Fatty Acid Metabolism after Silencing of DcTPS1

**Figure 7:**
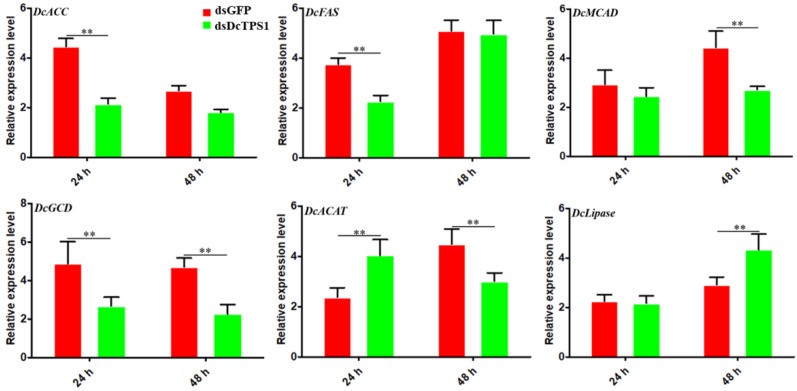
Effects of RNAi on key genes in *Diaphorina citri* fatty acid metabolism pathway. The ds*GFP* treatment group was used as a control. The mean expression level represents three biological replicates. The asterisks indicate significance differences by ** *p* < 0.01. The SPSS software was used to conduct statistical analysis.

Acetyl-CoA produced form glycolysis can be utilized to form lipids. In order to analyze the effect of *DcTPS1* on *D. citri* fatty acid metabolism, a total of six genes involved in synthesis and degradation of fatty acid were analyzed at 24 h and 48 h after knockdown of *DcTPS1*. The results suggested that two genes associated with fatty acid synthesis were significantly downregulated at 24 h after silencing of *DcTPS1*, whereas they had no significant change between ds*DcTPS1* group and ds*GFP* group at 48 h, including *acetyl-CoA carboxylase-like* (*DcACC*) and *fatty acid synthase-like* (*DcFAS*) (Figure 7). In addition, three genes involved in oxidative decomposition of fatty acids exhibited similar expression patterns, and they were downregulated at 24 h or 48 h after silencing of *DcTPS1*, including *medium-chain-specific acyl-CoA dehydrogenase* (*DcMCAD*), *glutaryl-CoA dehydrogenase* (*DcGCD*) and *acetyl-CoA acetyltransferase* (*DcACAT*). The expression level of *DcLipase* was significantly increased at 48 h after silencing of *DcTPS1*, but it had no obvious change at 24 h (Figure 7).

### 3.6. Transcriptome Sequencing and Reads Assembly

After removing the redundant reads, a total of 51,460,996 (97.8%), 47,166,336 (97.6%), and 42,176,502 (97.9%) clean reads from the treatment groups (ds*DcTPS1*); 40,384,536 (98.0%), 49,969,160 (98.0%), and 42,794,132 (97.8%) clean reads from the control groups (ds*GFP*) were obtained. The raw reads from the six libraries were submitted to the sequence read archive (SRA) of NCBI (BioProject: PRJNA782056). The values of Q20 and Q30 were approximately 97% and 93%, respectively. The values of GC content in different samples were about 40% (Table S2). Furthermore, 41,276,108 (80.2%), 37,944,770 (80.4%) and 34,460,721 (81.7%) clean reads from the treatment groups (ds*DcTPS1*); 32,409,083 (80.3%), 40,246,014 (80.5%) and 34,339,514 (80.2%) clean reads from control groups (ds*GFP*) were successfully mapped to the *D. citri* genome (Table S3).

### 3.7. Identification of DEGs and Functional Prediction

**Figure 8:**
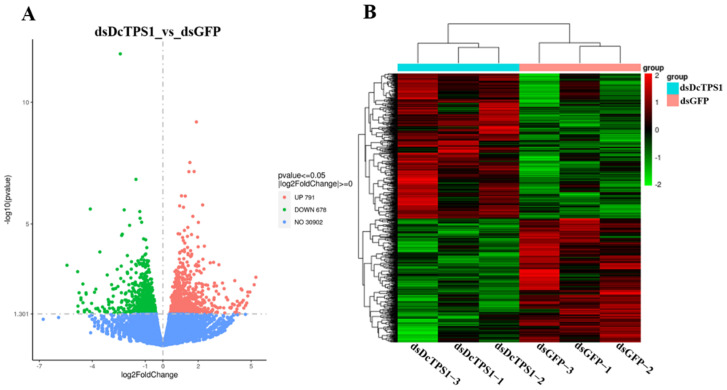
Identification and hierarchical cluster analysis of DEGs among different samples. (**A**) A volcano diagram for each gene. The red and green points indicate upregulated genes and downregulated genes, respectively. The blue point indicates no significant difference between ds*DcTPS1* and Dc*GFP* groups. (**B**) Hierarchical clustering of DEGs.

According to use the DESeq method, DEGs were identified between control groups (ds*GFP*) and treatment groups (ds*DcTPS1*). In total, 1469 DEGs were identified in ds*DcTPS1* groups compared with ds*GFP* groups, among which 791 DEGs were upregulated, and 678 DEGs were downregulated (Figure 8A; Table S4). The hierarchical clustering revealed that DEGs with similar expression patterns were clustered together, and further showed good repeatability among the three biological replicates (Figure 8B).

**Figure 9:**
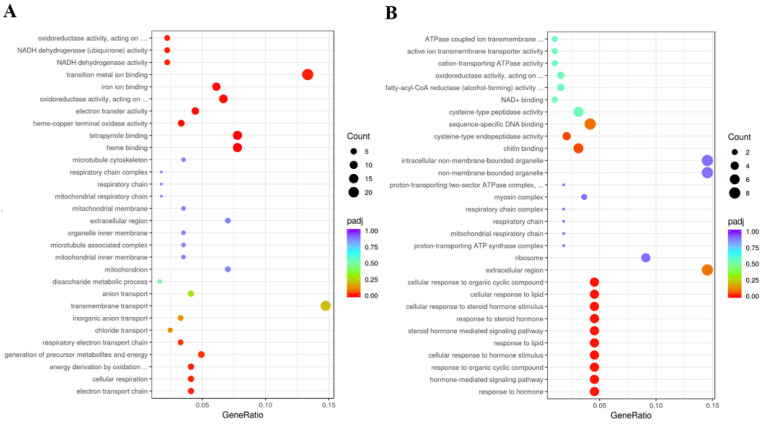
GO enrichment analysis of DEGs between ds*DcTPS1* groups and ds*GFP* groups. The sizes of the bubble indicate the number of DEGs enriched in the GO term. The color of the bubble indicates the Q value. (**A**) Upregulated DEGs. (**B**) Downregulated DEGs.

**Figure 10:**
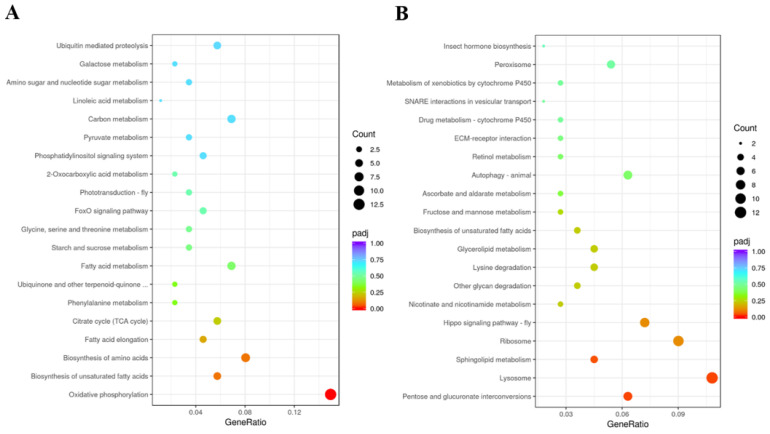
KEGG enrichment analysis of DEGs between ds*DcTPS1* groups and ds*GFP* groups. The sizes of the bubble indicate the number of DEGs enriched in the KEGG pathway. The color of the bubble indicates the Q value. (**A**) Upregulated DEGs. (**B**) Downregulated DEGs.

**Figure 11:**
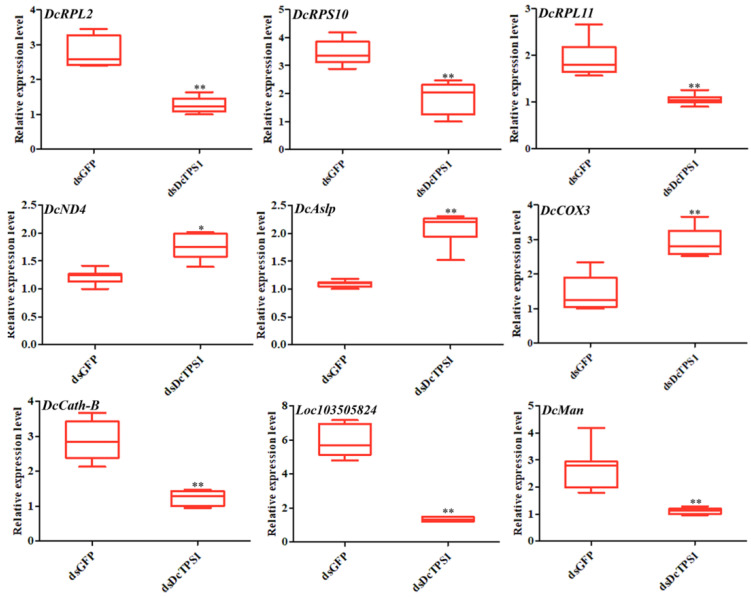
Validation of DEGs involved with the ribosome, oxidative phosphorylation and lysosome, respectively. The mean expression level represents three biological replicates. The asterisks indicate the significance differences by * *p* < 0.05 and ** *p* < 0.01. The SPSS software was used to conduct statistical analysis.

GO enrichment analyses suggested that upregulated DEGs were mainly involved in transition metal ion binding and transmembrane transport, and DEGs of downregulation were involved in chitin binding and sequence-specific DNA binding in the ds*DcTPS1*_vs_ds*GFP* groups (Figure 9; Table S5). KEGG enrichment revealed that upregulated DEGs were mainly involved in oxidative phosphorylation, and downregulated DEGs were associated with the lysosome and ribosome (Figure 10; Table S6). To further validate the expression levels of DEGs in the control groups and treatment groups, we selected nine DEGs involved with the ribosome, oxidative phosphorylation and the lysosome. The results showed that the express trend of nine genes remained consistent between qPCR and transcriptome data (Figure 11). A total of three DEGs related to ribosome were downregulated after silencing of ds*DcTPS1*, including *39S ribosomal protein L2* (*DcRPL2*), *40S ribosomal protein S10* (*DcRPS10*) and *60S ribosomal protein L11* (*DcRPL11*). A total of three DEGs related to oxidative phosphorylation were upregulated after silencing of ds*DcTPS1*, including *NADH-ubiquinone oxidoreductase chain 4* (*DcND4*), *ATP synthase lipid-binding protein* (*DcAslp*) and *cytochrome c oxidase subunit 3* (*DcCox3*). Additionally, three DEGs associated with lysosome were downregulated after silencing of ds*DcTPS1*, including *cathepsin B-like cysteine proteinase* (*DcCath-B*), *uncharacterized LOC103505824* (*Loc103505824*) and *alpha-mannosidase At3g26720* (*DcMan*).

## 4. Discussion

Trehalose is the principal sugar circulating in the hemolymph of most insects and is synthesized in the fat body. The stored energy reserve in the form of trehalose is hydrolyzed by trehalase to meet the energy demands for flight and development [6,35]. Chitin is a polymer of *N*-acetyl glucosamine that forms the protective exoskeleton of all arthropods and is replaced during growth and development [36]. The insect chitin biosynthetic pathway starts with trehalose and involves several key enzymes [2]. Therefore, accurate regulation of trehalose concentration is crucial for normal growth and development of insects. In insects, trehalose forms the major hemolymph sugar and is synthesized in a way that involves two enzymes, including trehalose-6-phosphate synthase and trehalose 6-phosphate phosphatase [37]. In the current study, a *TPS* gene was identified from the *D. citri* genome database. Bioinformatic analysis revealed that *DcTPS1* encoded a total of 833 amino acids (Figure 1). In previous research, Liu et al. also identified a *TPS* gene from *D. citri* which encoded a protein of 594 amino acid residues [15]. Tang et al. revealed that the insect *TPS* gene encoded an 820–850 amino acid protein with two conserved domains—TPS and TPP—corresponding to *OtsA* and *OtsB* genes in yeast [38]. Additionally, phylogenetic tree analysis suggested that DcTPS1 kept a close relationship with hemipteran insects, including *B. tabaci*, *A. pisum* and *N. lugens*. Interestingly, we also found that DcTPS1 had a close relationship with DcTPS reported by Liu et al. [15]. Therefore, we considered that DcTPS1 belonged to a *TPS* gene in *D. citri*. The number of *TPS* genes varies among various insects. In *Blattella germanica*, a total of two *TPS* genes were cloned, including *BgTPS1* and *BgTPS2* [39]. In *N. lugens*, a total of three TPS genes were identified, and these *TPS* genes had been found to encode proteins with two conserved TPS and TPP domains. However, we did not find *TPS2* or *TPS3* homolog sequences from the *D. citri* genome and transcriptome databases.

Insects store energy reserves in the form of glycogen and triglycerides in the adipocytes, the main fat body cell [40]. Furthermore, insect fat body is an important tissue for production of trehalose [41]. In this study, *DcTPS1* had a high expression in the midgut and wing, while it had a low expression in the fat body. Most insects express the *TPS* gene mainly in the fat body, including *Helicoverpa armigera*, *N. lugens* and *Bactrocera minax* [2,37,42]. We speculated that *DcTPS1* was mainly involved in trehalose synthesis in the midgut. High expression of *TPS* genes in the midgut was also found in some other insects. In *Leptinotarsa decemlineata*, *LdTPS* showed a high expression level in the fat body, foregut and hindgut, involving in the synthesis of trehalose [10]. Furthermore, we also considered that midgut tissue might have been doped with fat body during the extraction process. In addition, we also found that *DcTPS1* exhibited a high expression at the egg, fifth-instar nymph and adult stages. In *D. citri*, *DcTPS1* expression level in female adults was significantly higher than that of male adults. In previous report, Liu et al. also found that DcTPS1 exhibited a high expression in the *D. citri* adult stage [15]. Based on the high expression of *DcTPS1* in the egg and female adults, we speculated that *DcTPS1* might be involved in reproduction of *D. citri* eggs. In *D. citri*, the fifth-instar nymph stage is a critical period that involves progressing from the nymph stage into adult [43]. After molting of nymph, *D. citri* adults are required to synthesize more trehalose to maintain flight and bounce. Moreover, during the *D. citri* molting period, synthesis of more trehalose promotes chitin formation to maintain the rigid structure of new cuticle. Interestingly, we also found that *DcTPS1* had a high expression in the *D. citri* egg. We speculated that *DcTPS1* might be involved in chitin synthesis on the embryonic cuticle of *D. citri*. In *Rhodnius prolixus*, reduction of chitin synthase gene transcripts by RNAi significantly affected chitin deposition and eclosion of the first-instar nymph [44].

RNAi is now widely used as a useful tool for discovering or validating gene functions. At 24 h and 48 h after ingestion of ds*DcTPS1*, the relative expression levels of *DcTPS1* were significantly downregulated in treatment groups compared with control groups (ds*GFP* treatment), which indicated that *DcTPS1* was effectively silenced. Furthermore, the mortality and malformation rates increased after silencing *DcTPS1*, and the fifth-instar nymphs could not completely molt and die. From the phenotype, we found that *D. citri* wing exhibited curl, and abdominal cuticle could not completely molt after treatment with ds*DcTPS1*. Our results are similar to those previously reported by Liu et al. [15]. In *Tribolium castaneum*, silencing *TPS* genes lead to molting deformities and high mortality rates via regulation of gene expression in the chitin biosynthesis pathway [11]. Yang et al. revealed that silencing *N. lugens TPS* genes induced insects displaying aberrant phenotypes [45]. In addition, we also found that knockdown of *DcTPS1* gene reduced the expression levels of chitin metabolism-related genes. Therefore, we considered that inhibition of *DcTPS1* gene disrupted *D. citri* chitin synthesis, resulting in abnormal phenotypes.

Trehalose represents the main hemolymph sugar in many insects, which is an energy source that meets the demands of flight muscles and other energy-consuming organs [35]. In these energy-consuming organs, the energy is ultimately derived from lipid. The relative expression levels of two fatty acid metabolism-related genes were significantly downregulated at 24 h after silencing of *DcTPS1*, including *DcACC* and *DcFAS*. Acetyl-CoA carboxylase (ACC) is a major rate-limiting enzyme of fatty acid biosynthesis; its product, malonyl-CoA, also contributes to the regulation of fatty acid oxidation and elongation [46]. Fatty acid synthase (FAS) is a multifunctional enzyme involved in the formation of fatty acids [47]. In a previous report, Shi et al. revealed that *L. decemlineata TPS* RNAi survivors consumed a greater amount of foliage. On the contrary, silencing of the *DcTPS1* gene lead to a smaller polypide. The results indicated that inhibition of *DcTPS1* disrupts the synthesis of fatty acid. According to transcriptome sequencing, GO enrichment analysis showed that downregulated DEGs were significantly involved in chitin metabolism. KEGG enrichment analysis showed that upregulated DEGs were significantly involved in oxidative phosphorylation. In biological cells, oxidative phosphorylation is primarily involved in the synthesis of ATP and is also associated with the oxidation of NADH. We found that knockdown of the *DcTPS1* gene increased the expression levels of DEGs associated with oxidative phosphorylation. Therefore, we speculated that a reduction in *DcTPS1* expression level significantly suppress energy metabolism. When energy synthesis is insufficient, *D. citri* will activate oxidative phosphorylation through its negative feedback mechanism. Additionally, DEGs involved with the ribosome and lysosome were significantly downregulated after silencing of *DcTPS1*. Lysosomes play a central role in the degradation of extracellular and intracellular macromolecules [48]. The expression level of *D. citri cathepsin B* (*DcCath-B*) significantly decreased after inhibition of *DcTPS1*. The results indicated that knockdown of *DcTPS1* gene inhibited protein hydrolysis.

## 5. Conclusions

A *TPS* gene was identified from *D. citri* genome database. *DcTPS1* showed high expression in the midgut and fifth-instar nymph stage. Additionally, silencing of *DcTPS1* led to an abnormal phenotype. Knockdown of *DcTPS1* significantly reduced the expression levels of chitin metabolism-related genes and fatty acid synthesis-related genes, while the expression levels of fatty acid decomposition-related genes were downregulated. Furthermore, transcriptome sequencing analysis revealed that DEGs involved in oxidative phosphorylation were significantly upregulated, whereas DEGs attributed to the lysosome and ribosome were downregulated.

## Data Availability

All published data are available upon formal request.

## Supplementary Materials

The following are available online at https://www.mdpi.com/article/10.3390/insects12121074/s1, Figure S1: Multiple sequence alignment of the conserved domain of the TPS1 from four insect species, Table S1: Primers used in this study, Table S2: A summary of the transcriptomes in the different treatments in *D. citri*, Table S3: A summary of reads mapped to *D. citri* genome in different treatments, Table S4: Identification of differentially expressed genes in different treatments, Table S5: GO enrichment analysis of DEGs, Table S6: KEGG enrichment analysis of DEGs.
