# Supplementary material for: Functional Characterization of a Trehalose-6-Phosphate Synthase in Diaphorina citri Revealed by RNA Interference and Transcriptome Sequencing
Source: Insects. 2021 Nov 30;12(12):1074. doi: 10.3390/insects12121074 (PMC8709273; doi:10.3390/insects12121074)

Figure S1. Multiple sequence alignment of the conserved domain of the TPS1 from four insect species, including *Diaphorina citri* (MZ888936), *Acyrthosiphon pisum* (XP_001945523), *Bemisia tabaci* (XP_018916964) and *Nilaparvata lugens* (ACV20871). Signature motifs (HDYHL and DGMNLV) unique to TPS was presented with red frame.

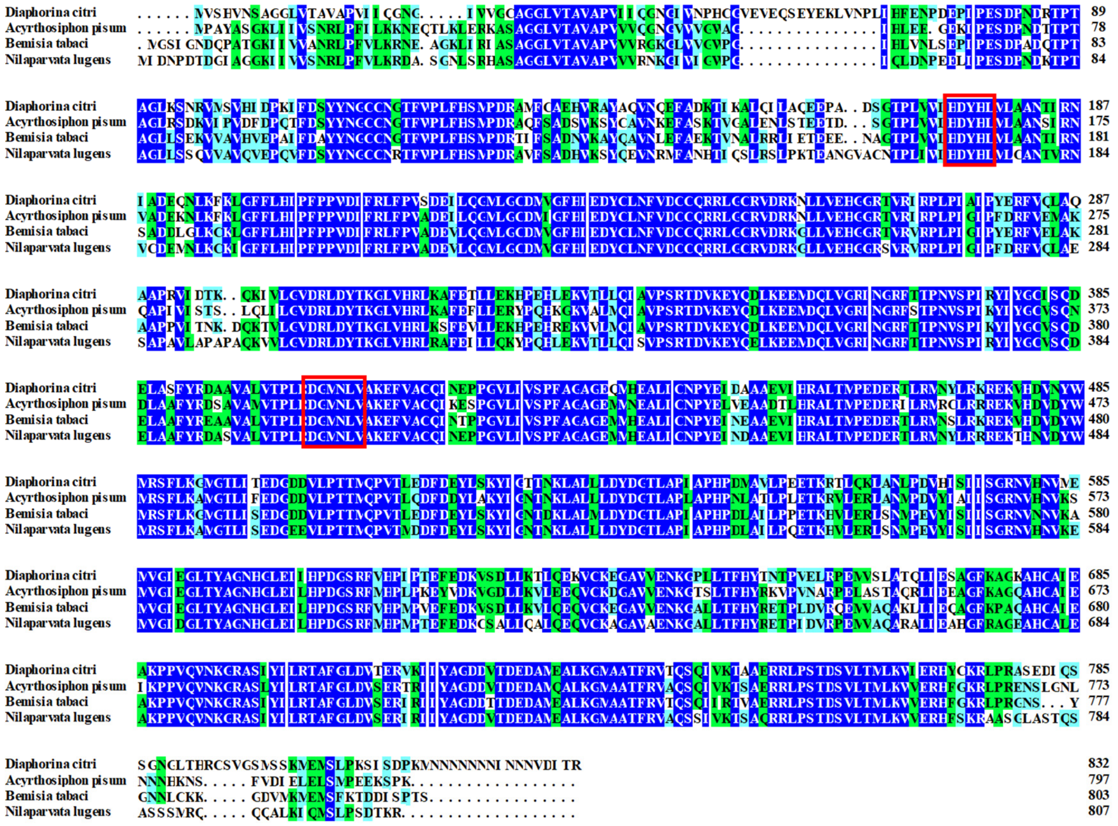

Supplement: Supplementary file 1 [file insects-12-01074-s001.zip › Supplementary File/Figure S1.pdf]
