# Supplementary material for: Functional Characterization of a Trehalose-6-Phosphate Synthase in Diaphorina citri Revealed by RNA Interference and Transcriptome Sequencing
Source: Insects. 2021 Nov 30;12(12):1074. doi: 10.3390/insects12121074 (PMC8709273; doi:10.3390/insects12121074)
Supplement: Supplementary file 1 [file insects-12-01074-s001.zip › Supplementary File/Table S1.pdf]

Table S1. Primers used in this study.

Genes	Sequences	Purpose
DcTPS1	F: GACATTTTCCGACTTTTCCCC<br>R: TTGCGGTCTACACGACAGCC	
DcCHS	F: TCAGCATGGCGGGTTAAG<br>R: CTCCGCGGAATGACATGAATA	
DcCHT	F: CGTGTATGCCTTTGCCGAT<br>R: TCCTTGAGTCTGACCAACCACC	
DcNAG	F: GTGGGAGTGGATGTGTTCGG<br>R: GCTTCAACTTGGACCAGAATAAAA	
DcTre1-1	F: AAATCATCACCAAGGACAATCAA<br>R: GAAGAGCCACTTTTCAGCCAG	
DcTre1-2	F: AAGCCAATGCTGGGAATCG<br>R: GGTCTGTGAAATCAGGGGGG	
DcTre2	F: CAAGGAATGGTTGGAGGCTGT<br>R: GCGGAGATAGGTTACTGGGGT	
DcHK	F: GACTTTCTGGCGGCGTGTATC<br>R: TGGTTGTCTTGTCGTCTGTGGA	
DcG6PI	F: TGCTGTGGGACCAAGAGTTCAT<br>R: ACTTTGCCCGCATTTGTAGATAG	
DcGNPNA	F: CGATTATGACCACGGGTTCCT<br>R: ACTTCACCAGAACGGCAATCA	
DcGFAT	F: TGAGCATTCACAGACTCCGCA<br>R: GCGGATTTCGGGAATGTAGTC	
DcUAP	F: AAGCCTGATGCTGTAGGAAATG<br>R: GCACAAGGAATGAATGACCAAT	
DcPAGM	F: GACAACTCAGGGGAAACTCAACT<br>R: GCAATACGGTCTCCATCCAACA	
DcACC	F: CCTTCCCGTGTCCACTCCTTA<br>R: TACGAAGTTGAGGACCTGAGCA	
DcFAS	F: GAAGGTTATCCCCTCTCCCAAG<br>R: CAGGAGGTTGTTGGTGTGGTATT	RT-qPCR analysis
DcMCAD	F: CTGTTCAGGTGTTTGGAGGCA<br>R: GGGTTACTTTGGTATGTTGGGG	
DcGCD	F: GGACCAGATGCTCCTTCTTGCTA<br>R: AGTTGTGGGATTTGGTTGGC	
DcACAT	F: CAGAAGGGAGTTGCCTCTATTTG<br>R: CGATAAACCAAAGTGTCCTCTCC	
DcLipase	F: CCCAACAGGATTCCGTGACTAA<br>R: GAACGGTCACATTACCAATAGGG	
DcRPL2	F: GCGAGTCATACAAATCATCCCA<br>R: ACACAGCATACAATGGAGCCC	
DcRPS10	F: GAAATCCAAAGGCTGGGTGA<br>R: TGTTGTGCGGGCTTGTCTCT	

DcRPL11	F: GCTGTAGACCCTGCCAAACC	
	R: AGGATTTCTTCTGCCTTTGCTC	
DcND4	F: ACTTCATTTATGGCTTCCTCGTG	
	R: GAACAAAACAAACACCAGTCAAAA	
DcAslp	F: GCTGTGAGCCGAGACATTGAC	
	R: GAAAGCGAACAACAACAGGAAA	
DcCOX3	F: ATCAGAGACTCAGTTTACGGAAGAAG	
	R: AATCATACTACATCTACAAAGTGCCAAT	
DcCath-B	F: GAGGCAAGAAAAAGACCACCA	
	R: GTGGGTTCCTTCAGGTATGGTT	
Loc103505824	F: TTACAGTGAAGGTCAACCACGAATA	
	R: TGGTCCAGTGATGTCCTTTCTCTAC	
DcMan	F: ACCAGACAGACATCGGCAACA	
	R: AGCGGTAGGTTCAGTGAGCGT	
GAPDH	F: CATGGCAAGTTCAACGGTGA	
	R: CGATGCCTTCTCAATGGTGG	
ds-*DcTPS1*	F: GGATCCTAATACGACTCACTATAGGGCGGAAGAGGGAGAAA	
	R: GGATCCTAATACGACTCACTATAGGTTCAACAGGTCGCTCACT	dsRNA synthesis
ds-*GFP*	F: GGATCCTAATACGACTCACTATAGGCAGTGCTTCAGCCGCTACCC	
	R: GGATCCTAATACGACTCACTATAGGACTCCAGCAGGACCATGTGAT	

The black underline indicates T7 promoter.
